# Development of an HRM-qRT-PCR platform for fast and cost-effective genotyping of infectious bronchitis virus in Egypt

**DOI:** 10.1038/s41598-026-45311-9

**Published:** 2026-04-10

**Authors:** Asmaa Ali, Abdelbary Prince, Samira H. Aljuaydi, Aya M. Yassin, Rabab Khalifa, Shahin Dardir, Said Z. Moussa

**Affiliations:** 1 Biochemistry and Molecular Biology Department, Faculty of Veterinary Medicine, Cairo University, Giza, 12211 Egypt; 2 Cairo poultry company (CPC) laboratory Division, Cairo, Egypt; 3 Technical office and Laboratories Department, Cairo Poultry Corporate, Giza, Egypt

**Keywords:** High-resolution melting analysis, Sequencings, Infectious bronchitis virus, Spike 1 gene, Nucleocapsid, 3′UTR, Biological techniques, Biotechnology, Diseases, Genetics, Microbiology, Molecular biology

## Abstract

Avian infectious bronchitis virus (IBV) is a highly transmissible virus that causes respiratory, reproductive, and renal syndromes, resulting in significant economic losses for the global poultry industry. This study aimed to evaluate the application of high-resolution melting analysis (HRM) as a novel, efficient, and cost-effective tool for the rapid diagnosis and genotyping of Egyptian IBV strains. The investigation focused on the N gene and the 3’ untranslated region (3’ UTR). HRM curves were generated from 435 bp PCR products obtained from 22 IBV-positive avian samples. The purified PCR products, which amplified the 3’ UTR, were sequenced and subsequently compared with analogous S1 gene sequences to differentiate between vaccine strains and field strains, specifically the Egyptian strain 4/91 (GI-13), variant II (GI-23), and the classic strain (GI-1). The high-resolution melting curve analysis (HRM) successfully distinguished vaccine reference strains (Ma5, IB-Primer, 4/91, and Variant II), while the IBV field strains were categorized into three distinct pattern clusters based on the similarity of their melting curves, which were confirmed by S1 gene sequences. Both strains 4/91 and variant II exhibited a deletion of about 15 nucleotides at the terminus of the N-gene compared to classic strains. Closed-tube PCR combined with HRM-curve analysis provides a fast and reliable technique for genotyping IBV strains, including IBV vaccine strains and IBV field strains.

**Supplementary Information:**

The online version contains supplementary material available at 10.1038/s41598-026-45311-9.

## Introduction

Avian infectious bronchitis virus (IBV) is a highly contagious respiratory virus that affects chickens, resulting in significant economic losses to the poultry industry, especially in Egypt. The disease is characterized by respiratory symptoms, such as gasping, coughing, and sneezing^1,2^. It also affects the reproductive system in laying birds, leading to reduced egg production and quality, as well as causing kidney damage in the case of nephropathogenic IBV strains^3,4^.

IBV belonging to the gammacoronavirus subfamily Coronavirinae and possesses a positive-sense single-stranded RNA virus with a 27.6-kilobase genome^5^. The genome is flanked by 5’ and 3’ untranslated regions (UTRs) and is organized as 5’UTR-[Rep1a-Rep1b-S-3a-3b-E-M-4b-4c-5a-5b-N-6b]−3’UTR^6^. However, some strains lack the accessory genes 4b, 4c, and 6b^7–9^. Gene 1, which spans approximately 20 kb and approximately two-thirds of the genome is located at the 5’ end and encodes the viral replicase polyproteins that are cleaved into 15 non-structural proteins important for viral replication. The spike (S) glycoprotein, the largest and most variable IBV protein, is encoded by gene 2 and is cleaved by host proteases into the S1 and S2 subunits^7–10^.

The untranslated regions (UTRs) of the IBV genome are important in the transcription and replication process of viral RNA^11^. The 3’UTR is located downstream of the nucleocapsid (N) gene, involved in initiating the synthesis of negative-strand RNA. It contains conserved as well as hypervariable elements. Comparative analysis revealed that this region gives extra information for strain identification and genotyping, complementing coding region analysis^16,17^.

Massachusetts (Mass) and H120 strains of vaccine, together with the D274, IBV variant I (IS/222/96, IS/251/96, and IS/64714/96) and variant II strains (IS/223/96, IS/572/98, IS/585/98, and IS/589/98), are common IBV strains in the Middle East^12,13^. By late 2009 and 2011, a distinct IBV Q1 strain had been reported in Jordan, Iraq, and Saudi Arabia. Diagnosis of IBV is generally based on the clinical signs, as well as the use of RT-PCR or quantitative real-time PCR (qRT-PCR). Although sequencing of the complete S1 gene or a specific segments is considered the gold standard method for IBV genotyping^14,15^. Because the S1 gene is highly diverse and evolves rapidly, depending on it alone can slow down outbreak investigation and control. Molecular analysis that includes both the S1 and N gene regions can provide a more consistent genetic picture of circulating IBV strains, guiding vaccine selection towards a broader spectrum of protection^14,15^.

As an advanced mutation-scanning technique, high-resolution melting (HRM) analysis delivers considerable savings in time and cost in comparison with alternative screening methods. This robust technique allows the detection of sequence variations for mutations by tracking fluorescence shifts that accompany the melting of denatured double-stranded DNA at elevated temperatures. The resulting melting plots allow straightforward discrimination between wild-type and heterozygous samples. Additionally, HRM analysis uses a closed-tube approach, enabling polymerase chain reaction amplification and subsequent analytical steps to be performed consecutively within the same well. This characteristic renders HRM analysis more practical compared to alternative scanning methodologies^18,19^.

As a result, the purpose of this research was to evaluate the application of HRM analysis as an effective, low-cost solution to the rapid diagnosis and genotyping of IBV in Egypt, targeting the N-gene and 3′UTR, offering a new method for monitoring viral strains and evaluating vaccine effectiveness in poultry flocks.

## Materials and methods

### Clinical information, samples collection, and processing

A total of sixty samples were gathered from various broiler and suspected layer flocks. Four commercially available live IBV vaccines were obtained from local markets in Egypt. The most widely used IBV vaccines are Mass-type, IB-Primer, 4/91, and IBV Variant II strains, which were used as reference strains for the collected samples, which originated from poultry flocks afflicted with clinical manifestations of the disease, including respiratory symptoms, egg production problems, and renal lesions that are associated with high mortality and morbidity rates (Supplementary 1).

### Viral RNA extraction

The procedure was performed as specified by the manufacturer, viral RNA was extracted from cystic oviducts, cloaca, tracheal swabs, kidneys, and IBV vaccine strains using an Easy spin^®^ RNA extraction kit (iNtron Biotechnology Inc.; South Korea).

### Screening for positive IBV samples by real-time PCR

IBV detection in collected samples and real-time reverse transcription-polymerase chain reaction confirmation of the virus’s existence (RT-PCR). The primer set utilized for amplification of the target genome was as follows^20^:

Real-time RT-PCR was performed using TransScript^®^II Probe One-Step qRT-PCR SuperMix. RT-PCR reactions were performed on one Rotor-Gene Q 5plex HRM Platform (Qiagen, CA, USA)—identification of Variant O2 from positive IBV samples by using Kylt^®^ IBV-Variant O2 Real-Time RT following kit protocol.

### High-resolution melting curve assay

#### cDNA synthesis

Complementary DNA (cDNA) was synthesized from IBV-positive samples, variant II (positive and negative), and vaccine samples using a commercial first-strand cDNA synthesis kit (Jena Bioscience, Jena, Germany), following the kit protocol. Briefly, 1 µl of random hexamer primers was added to 7 µl of extracted viral RNA and 2 µl of RNase-free water, and the mixture was heated at 70 °C for 5 min and then placed on ice for 5 min. Ten microliters of cDNA master mix containing reverse transcriptase, 1x reaction buffer, dNTP mix, and RNase inhibitors. The reaction was incubated at 42 °C for 10 min, followed by reverse transcription at 50 °C for 60 min, and enzyme inactivation at 70 °C for 5 min. The synthesized cDNA was stored at −20 °C until it was used for RT-PCR.

#### qPCR and HRM

On a Qiagen Rotor-Gene Q 5plex HRM Platform (Qiagen, CA, USA) with the QIAGEN Type-it HRM PCR Kit (Qiagen, CA, USA) according to the manufacturer’s instructions, HRM was performed on 22 qRT-PCR positive IBV samples detected from 60 screened samples. In total, 25 µl reactions were set up with 12.5 µl of 2× HRM PCR master mix and 0.2 µM of each of the forward and reverse primers (Supplementary 2). 5 µl of cDNA and nuclease-free water. Thermal cycling conditions were an initial activation step of PCR (5 min at 95 °C), 40 cycles of 95 °C for 10 s, 57 °C for 30 s, and 72 °C for 30 s, and a final elongation step of 60 s at 72 °C. Melting analysis was performed following initial denaturation from 65 to 95 °C, in 0.3 °C increments^21–23^.

HRM analysis software was used for genotype determination by assigning a reference IBV genotype (Mass, 4/91, or Variant II). Normalization of the data results in HRM Genotype Confidence Percentages (GCPs).

The isolate is assigned to a genotype by comparison with sequencing-confirmed reference strains included in each run. During assay validation, GCPs thresholds between 90% and 99% were evaluated using sequencing-confirmed reference strains of IBV. A 97% cutoff was selected based on complete concordance with Sanger sequencing and optimal separation of closely related genotypes within the S1 region. This method is consistent with previous IBV HRM studies^22,23^, where genotype confidence ranges were statistically defined by calculating the mean GCPs and standard deviation, and the lowest 5% of values were excluded using a standard of −1.645 × SD to avoid misclassification. In our study, the 97% threshold excluded borderline melting curve variation, thus ensuring reliable genotype identification.

Nine isolates were selected for sequencing after HRM analysis: three vaccine strains (Ma5, 4/91, and Variant II) and six field strains. Vaccine strains and field isolates were sequenced for partial N-gene and 3′UTR. Six field isolates were also sequenced for the entire S1 gene to confirm the IBV genotype.

#### Sequencing validation

Selected HRM products were sequenced using Sanger sequencing using (All-F, Dell-R) primers targeting the partial N-gene and 3′UTR of the vaccine and field IBV strains^23^. For the S1 gene, nucleotide sequencing was performed on IBV field strains using the following primers: 5’-AAG ACT GAA CAA AAG ACC GAC T-3’ sense primer and 5’-CAA AAC CTG CCA TAA CTA ACAT A-3’ antisense primer targeting 1760 bp of the S1 gene^9^.

#### Bioinformatics

Chromas Pro version 1.5 beta, developed by Technelysium Pty., Tewantin, QLD, Australia, is utilized for processing and assembling sequence data. IBV sequences were quality assessed using FinchTV v 1.4.0 after assembly to guarantee data trustworthiness. The Basic Local Alignment Search Tool (BLAST) was used, a robust tool for finding sites of local similarity between sequences, to compare our results to those in GenBank. Muscle alignment was used because of its stellar reputation for accurately aligning datasets. Using the Tamura 3-parameter model, the genetic distances among the sequences were computed^24^, which takes into consideration the fact that nucleotide substitution rates are not uniform and that these rates might vary between sites. A graphical depiction of the genetic variation was provided using the neighbor-joining model, which uses the Tamura 3-parameter model to infer phylogenetic relationships. Bootstrapping, carried out in MEGA 11.0, was used to assess the robustness of the phylogenetic trees^25^, with 1000 replicates to assess the reliability of the inferred tree topologies. For enhanced visualization of phylogenetic trees, the ITOl software was utilized^26^, allowing for interactive tree visualization. In addition, a minimal spanning network was built using PopArt v.3.0, which was used to evaluate the haplotype divergence within the dataset. Finally, genetic divergence was examined by visualizing the genetic distance matrix using the statistical computer program R V4.2. This matrix is essential for comprehending the genetic links and diversity among the viral strains.

## Results

A total of 24 nucleotide sequences were analysed in this research. Among them, ten sequences were obtained from the Ma5 strain, 3 of which were vaccine sequences. From the variant II strain, six sequences were analyzed; one was a vaccine, and eight sequences were taken from the 4/91 strain (2 of which are vaccine sequences). All codon positions were included in the study, including the first, second, third, and noncoding areas. This inclusion ensures a complete portrayal of the genetic material. During sequence processing, the pairwise deletion option is checked to delete any unreliable sites from each sequence pair carefully. By removing any ambiguous or uncertain genetic information, this method improves the analysis’s accuracy. An extensive foundation for detailed genetic research was provided by the improved dataset, which was validated by employing 343 sites.

**Fig. 1:**
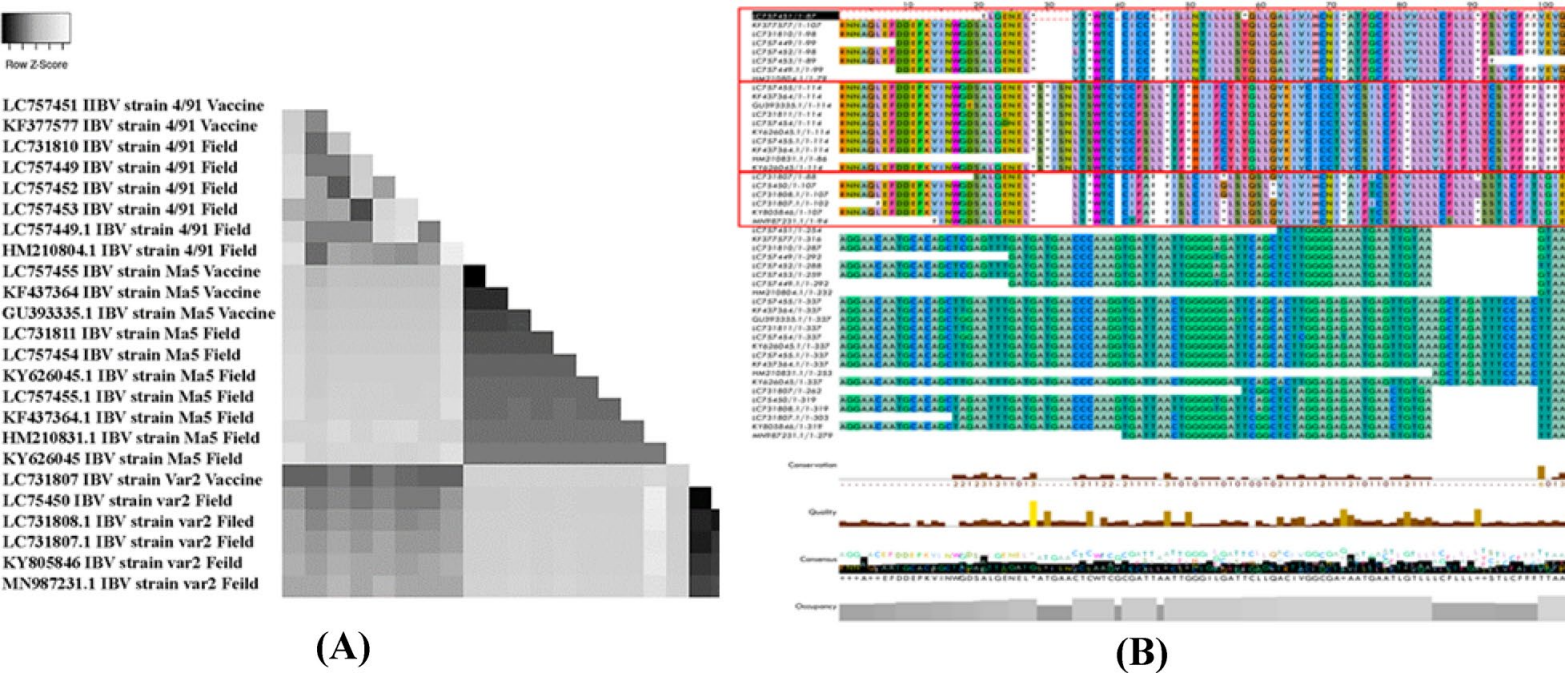
**A** Show distance matrix computed using Tamura 3-parameter model, and **B** Show multiple sequence alignment and deduced amino acid sequence of IBV isolates and selected reference from GenBank. 15 bp deletion in variant II and 4/91 strains at the N/6b junction compared to mass-type strains.

To better comprehend the genetic links and sequence changes, the Tamura 3-parameter model was deployed to compute the matrix of pairwise distances^27^. This model is preferable because it provides a more realistic evolutionary distance by taking into consideration variability across different sites and variable rates of nucleotide substitutions. A genetic distance matrix, as shown in Fig. 1A, provided light on the relative genetic distances of the studied strains.

Figure 1B shows a more in-depth visual depiction of the genetic structure of the three strains stated earlier by expanding on their respective amino acid and nucleotide sequences. Additionally, the sequences’ levels of similarity or divergence are shown via color coding. The sequence quality is shown at the bottom of the page as a bar graph. The proportion of bases with a Phred score of 20 or above is indicative of very high confidence in the sequencing’s correctness and is, therefore, a good indicator of quality. As shown, the translated protein is subjected to intense selection pressure, which causes an obvious change in the amino chain between different strains. In terms of donor sequence similarity, the Ma5 strain ranks highest, followed by the variant II strain, and finally, the 4/91 strain. This data suggest that the Ma5 strain appears more basal of the three strains, with the more evolved Ma5 and variant II strains being subsequent generations of the infection. Deletion of about 15 bp in both variant II and the 4/91 vaccine and field samples at the end of the nucleocapsid gene and the start of the 6b gene compared with the Ma5 vaccine (mass type), and this is reflected in the different melting curves.

Based on the present dataset, the Tamura 3-parameter model is the best for building a neighbor-joining tree^24^, due to its accuracy in depicting evolutionary distances between sequences, taking into consideration things like variable base frequencies and substitution rates. When compared to simpler models, it depicts evolutionary connections with greater subtlety.

Following the recommendation, bootstrap analysis was performed with 1000 replicates to bolster the phylogenetic analysis. This approach verifies the stability of branches, which increases the phylogenetic tree’s credibility. Branches with less than 50% support collapse since they are not reliable^27^. This way, only strong clades are shown. A clear and accessible depiction of the bootstrap support is provided graphically with colour-graded branches in the tree, which indicate the proportion of duplicate trees where distinct strains are grouped.

**Fig. 2:**
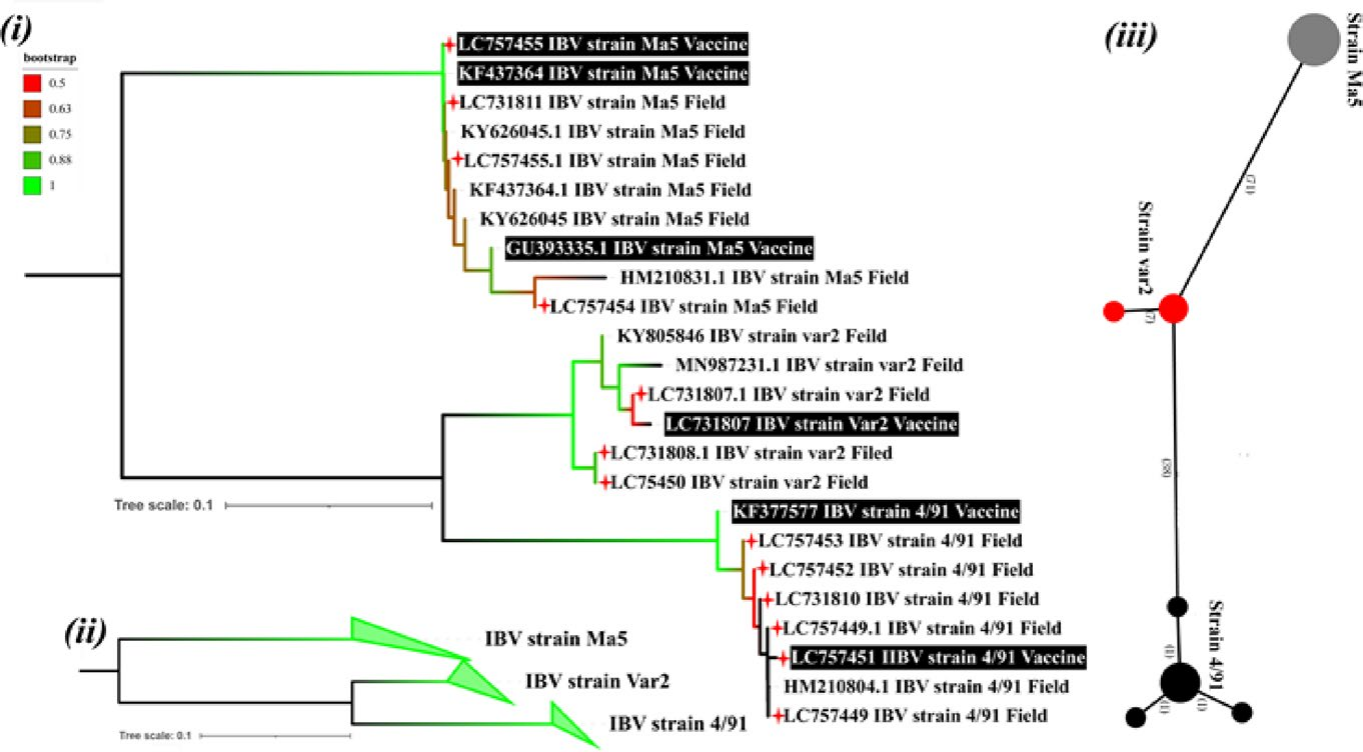
Phylogenetic network based on the IBV nucleocapsid (N) gene and 3′untranslated region (3′UTR). **i** A neighbor-joining tree illustrates the genetic relationships between field isolates and reference vaccine strains. **ii** The clustering of IBV strains into distinct genotypic groups is shown by a collapsed NJ tree. **iii** The genetic relatedness between field isolates and vaccine strains is shown by a minimum Spanning Network.

A matrix of pairwise distances, computed using the Tamura 3-parameter model, was used to obtain the tree. This all-encompassing method guarantees a solid and well-supported evolutionary tree by using 24 nucleotide sequences (Fig. 2i & ii).

In addition, the present study delves into the variety of stereotypes and how they differ across the studied strains by analyzing the minimal spanning network for the dataset. High genetic diversity was observed through nucleotide diversity assessed with a pi-value of 0.494645. With 89 of the 92 segregating sites being parsimony informative. We can see that there are relevant evolutionary markers present. Furthermore, the non-randomness in the allele frequency distribution was suggested by Tajima’s D statistic, which was 2.8549 with a p-value of 0.000592118. This might be a result of evolutionary factors like selection or expansion (Fig. 2 iii). This comprehensive statistical analysis provides a deeper understanding of the genetic structure and evolutionary dynamics of the studied strains.

### Molecular characterization (qRT-PCR/HRM and S1 sequencing)

To identify both vaccine and field strains of IBV, a total of 22 field samples were analyzed with reference vaccines (Ma5, IBV primer 4/91, and 1212B Variant II). Screening was first performed using qRT-PCR targeting part of the N gene region and the 3′UTR, followed by HRM (High Resolution Melting) curve analysis.

HRM analysis was carried out on a Rotor-Gene Q 5plex HRM platform. PCR products were gradually heated from 65 °C to 95 °C at increments of 0.3 °C/s, and conventional melting curves were generated automatically. The resulting HRM profiles typically showed two peaks, reflecting amplicon-specific melting behavior. Normalization regions were applied between 80.43 and 81.45 °C and 84.03–85.06 °C to ensure consistency across samples.

Genotyping was achieved by selecting representative reference strains (Mass, 4/91, IBV primer, and Variant II) and allowing the software to auto-assign sample genotypes with associated confidence percentages (GCPs). While no confidence threshold was applied, replicates with < 97% CPG were considered distinct and potentially indicative of a different genotype.

**Fig. 3:**
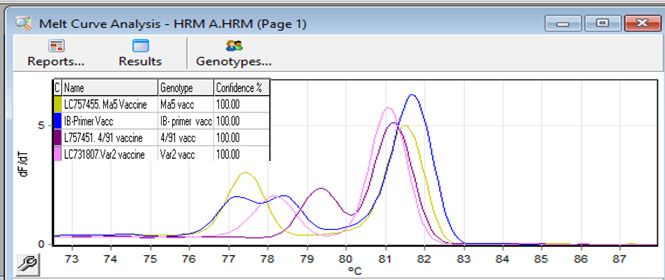
HRM Normalized graph of the vaccine strain.

The melt curve analysis presented in (Fig. 3) is the result of a HRM experiment in which nucleic acid sequences were differentiated according to their respective melting temperatures. The graph illustrates the relationship between temperature (y-axis) and the derivative of fluorescence (dF/dT) on the y-axis; each peak corresponds to a distinct sequence melt profile. There are three discernible peaks, each of which corresponds to a unique vaccine strain designated as “MA5 vaccine,” “4/91 vaccine,” “IB-Primer vaccine,” and “Variant II vaccine.” The distinct nucleic acid sequences of the vaccination strains are demonstrated by the varying positions and forms of the peaks, which dissolve (melt) at different temperatures, thereby revealing the existence of genetic variety among the strains. As a feature of its GC content and length, the melting temperature of the particular amplicon under examination can be deduced from the temperature at which a peak arises.

The HRM analysis findings of the RT-qPCR for investigated strains are briefly shown in Table 1, comprised of 4 vaccinations and field isolate specimens; the strains are designated by their respective GenBank accession numbers. The two peaks of each sample’s melting temperature (Tm) are presented; these peaks provide information regarding the genotypes and nucleic acid sequences. The Mass and Variant I genotypes have discernible Tm values, while the Mass strain demonstrates comparatively lower Tm values for Peak I in contrast to Variant I. The genotype classification’s reliability is denoted by the confidence percentages, with the majority of samples exhibiting a confidence level exceeding 80%. Based on their close relationship with the vaccine strains, the field isolates derived from the vaccine genotypes.

**Table 1:** Correlation between HRM Profiles and S1 gene sequencing of vaccine and field-Derived IBV Isolates” respective access number in the GenBank, classification of genotype and Tm, and confidence %.

Sample Code	Accession Number(S1)	Identity score	Accession Number (*N* &3′UTR)	Peak I Tm	Peak 2 Tm	Genotype	Confidence% (GCPs)
Ma5 vaccine	KY626045	100%	LC757455	77.45	81.5	Massachusetts	100%
4/91 vaccine	KF377577	100%	LC757451	79.34	81.2	Variant I	100%
Variant II vaccine	KU979007	100%	LC731807	78.2	81.05	Variant II	100%
Field D1	LC760462	90.53%	LC731808	79.05	80.91	Variant II	92.7%
Field D2	LC760463	87.75%	LC731809	79.16	80.99	Variant II	88.85%
Field D3	LC760464	95.3%	LC757452	79.34	81.2	4/91 (Variant I)	98.5%
Field D4	LC760465	95,69%	LC731810	79.34	81.2	4/91 (Variant I)	99.4%
Field D5	LC760466	84.01%	LC757453	79.41	81.3	4/91 (Variant I)	96.53%
Field D6	LC760467	93.0%	LC731811	77.36	81.44	Massachusetts Ma5	93.78%
Field D7	NT		LC757454	77.45	81.80	Massachusetts H120	82.89%
Field D8	NT		LC75450	78.50	80.90	Variant II	77.4%

Data is presented as frequency (%).

### Interpretation of S1 sequencing and HRM results

**Fig. 4:**
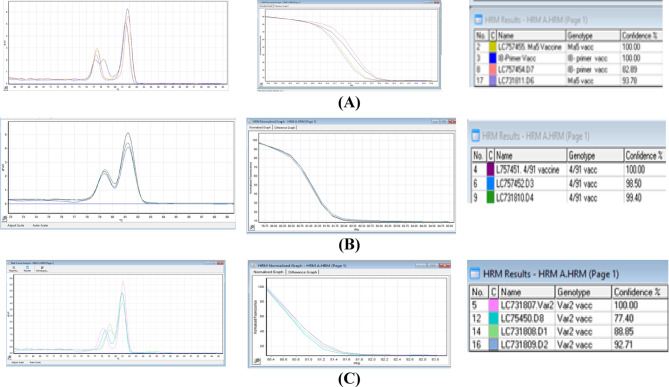
**A** HRM Normalized graph of Ma5 vaccine versus Field sample, **B** HRM Normalized graph of 4/91 vaccine versus Field sample, and **C** HRM Normalized graph of Variant II vaccine versus Field IBV strain.

**Fig. 5:**
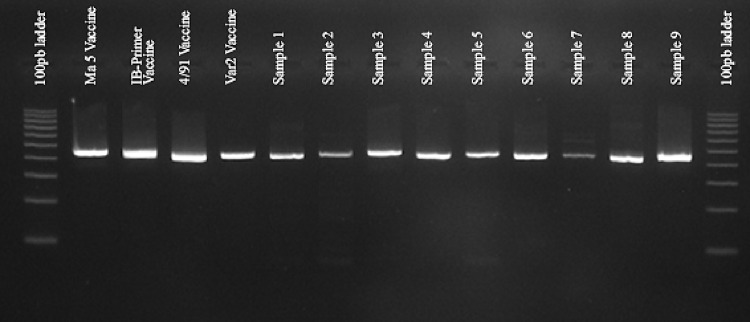
HRM-PCR amplicons from IBV vaccine strains and field samples are displayed in full-length uncropped agarose gel electrophoresis. Lane arrangement: 100 bp ladder, Ma5, IB-Primer, 4/91, IBV variant II vaccines, Samples 1–9 and 100 bp ladder.

The HRM analysis results, in Fig. 4, were used to distinguish genetic variations between strains by observing the variations in their DNA melting temperatures. The graphs represent comparisons between field isolates versus their matched vaccination strain. The outcomes demonstrate the dependability of characterizing strains with melting curves as opposed to a time-consuming and costly sequencing technique. Two distinct peaks were formed for each IBV vaccine and field sample in the standard melt curve analysis, which varies by genotype. A single stained band was observed upon agarose gel electrophoresis of the PCR product (Fig. 5). Proving that the presence of extra peaks in the PCR was not caused by other amplifications.

When combined with S1 gene sequencing, the HRM assay clearly grouped the examined strains into three major genotypes: Massachusetts (Ma5), 4/91 (Variant I), and Variant II. The HRM GCPs for all related IBV strains (based on an S1 gene identity ≥ 95% and a 3′UTR identity of ≥ 97%) indicating the same sequence^22^.

Among the field isolates, two isolates (D1 and D2) revealed a melting curve pattern similar to the Egyptian Variant II reference strain. This finding suggested that the Egyptian variant II field strains share a close sequence with the variant II vaccine, but they are not vaccine strains. Three samples (D3, D4, D5) grouped with the 4/91 genotype (Fig. 4A). The first two samples corroborated the S1 sequence and were comparable to the 4/91 vaccine; while the third sample S1 sequence shared only 84% identity with the vaccinal strain, it showed 96.53% identity in the N-gene and 3′UTR regions by HRM. This suggests a mismatch between the sequencing results and HRM: S1 sequence analysis identified the isolate as a field strain associated with 4/91, whereas HRM suggested similarity to the vaccine strain. One isolate (D6) grouped with the Massachusetts genotype (Fig. 4C), showing 93% sequence identity and 93% HRM confidence; this indicates the sample is a field strain, not the Ma5 vaccinal strain.

## Discussion

IBV continues to be one of the most important threats to the poultry industry globally^28,29^. The effect is not only important for bird health but also has considerable economic aspects, as it can lead to a rapid decline in egg production, lower egg quantity, slower broiler growth, and sometimes higher mortality^3,4,29^. For the poultry sector, this means direct financial losses, to which costs for veterinary treatments and biosecurity measures and vaccination campaigns must be added^30,31^. Understanding the genetic diversity of IBV is a major key to its control. Many strains with varying levels of tissue tropism and pathogenicity are produced via rapid evolution. Vaccines that work against one strain won’t work against another. Identifying the strain causing an outbreak is essential. By genotyping, IBV can help in monitoring the emergence of new variants, identifying the optimal management strategy, and differentiating vaccine from field strains^31,32^. Although S1 gene sequencing has long been regarded as the gold standard for genotyping, quicker molecular techniques like HRM analysis are becoming more and more useful during epidemics. These techniques make it possible to identify and characterize circulating strains in a timely manner, allowing for prompt action to stop their spread and minimize financial losses^33^.

In this study, we combined phylogenetic reconstruction, HRM analysis, and comparative sequence analysis to gain a detailed understanding of the genetic relationships among the Ma5, Variant II, and 4/91 IBV lineages currently circulating in Egypt. Together, these approaches provide a coherent view of how these lineages are evolving, how they relate to one another, and how molecular tools can support rapid and accurate field detection.

Phylogenetic and network analyses provided a clear overview of the genetic relationships among the Ma5, variant II, and 4/91 IBV strains included in this study. The neighbor-joining tree revealed a clear grouping of vaccine and field isolates into three major groupings that corresponded to the 4/91, variant II, and Ma5 lineages (Fig. 2i). The isolates in the Ma5 cluster showed considerable genetic similarity, as evidenced by the cluster’s greatest bootstrap support. Indicating strong genetic similarity among isolates within this group. The Variant II isolates formed a separate intermediate cluster, while the 4/91 isolates grouped distinctly and showed greater divergence from Ma5. The gradient in Bootstrap colors further reflected the relative confidence in branch placements, with Ma5 exhibiting the most stable topology.

The simplified, collapsed NJ tree emphasized the broader evolutionary relationships. This representation clearly illustrated that Ma5 branches earlier than the variant II and 4/91 lineages (Fig. 2 ii), supporting the interpretation that Ma5 may represent the most ancestral form of the three. Meanwhile, variant II occupies an evolutionary middle position, consistent with its intermediate sequence similarity. The minimum spanning network reinforced these findings by mapping mutational steps between the three groups (Fig. 2 iii). The Ma5 cluster appeared as the most cohesive, with fewer mutational steps among its members, whereas 4/91 displayed the greatest separation from Ma5, forming a distinct and more genetically diverse cluster. Variant II again bridged the genetic space between Ma5 and 4/91. These patterns align with the donor sequence similarity ranking, where Ma5 demonstrated the highest similarity, followed by variant II and then 4/91.

A key genetic feature contributing to these differences is the approximately 15-bp deletion detected in both variant II and 4/91 across vaccine and field isolates at the junction between the end of the N gene and the beginning of the 6b gene when compared with Ma5 (Mass type). This deletion likely explains the distinct melting curve profiles observed in HRM analysis, demonstrating the technique’s sensitivity to even small sequence variations. The ability of HRM to detect such differences highlights its value for rapid differentiation and characterization of IBV strains.

For HRM investigation, the IBV3’UTR hypervariable region was utilized since it appeared to be less susceptible to spontaneous mutation than the S1 gene^34^. Present methods for identifying IBV strains necessitate the extraction of the virus from infected tissues using SPF eggs to generate RNA of superior quality, which is essential for effective PCR and S1 gene sequencing^11,13^. By employing a small fragment for a PCR product that may be produced directly from infected tissue, thus eliminating the requirement for viral isolation, the methodology devised in this research is relatively more rapid. In fact, the overall process outlined in this document can be finished within a timeframe of less than five hours. This is far quicker than traditional DNA sequencing, which requires a few days (if the necessary equipment is easily accessible), not including the time required for further analysis and interpretation of the sequencing outcomes. The strategy outlined additionally offers cost advantages. The utilization of the Syto-9 dyes merely increased the expense of conventional PCR^18,21,22^.

Our study builds on this approach by applying HRM to Egyptian isolates and comparing the results with conventional S1 gene sequencing. The HRM curves were derived from 435-bp PCR products of multiple IBV strains and samples obtained from suspected flocks.

The HRM assay successfully clustered the investigated IBV samples into Massachusetts, 4/91, and Variant II genotypes, consistent with reference vaccine strains.

For almost all samples, HRM and S1 sequencing showed good agreement; one discrepancy was observed in sample D5. This isolate clustered with the vaccinal 4/91 strain in HRM analysis (N-gene/3′UTR) but was clearly identified as a 4/91-related field strain by S1 sequencing. Such differences arise because the N-gene and 3′UTR are relatively conserved, whereas the S1 gene is highly variable and under strong immune selection pressure. To overcome this limitation, HRM should be interpreted as a rapid genotyping screening tool rather than a stand-alone confirmatory method. Integrating HRM with S1 sequencing for selected isolates, especially those with borderline confidence percentages or epidemiological significance, would ensure accurate classification.

The study’s limitation was based on a limited number of samples, but the agreement between the HRM profile and sequencing results supports the reliability of the assay. Expanding the assay validation to larger sample sets from different regions of Egypt is planned as future work. The reference panel was limited, but it represents the most frequently used vaccine strain in Egypt. Future validation should include additional genotypes. Such as QX-like, Q1, and Field variant strains to further expand the diagnostic coverage.

## Conclusion

This study showed that a quick, accurate, and economical method for identifying and preliminary genotyping the IBV circulating in Egyptian poultry flocks is HRM analysis of the N-gene and 3′UTR. With outcomes that were generally in line with S1 gene sequencing, the technique effectively grouped field isolates into the three main genotypes (Massachusetts, 4/91, and Variant II). Crucially, although HRM offered the ability to screen quickly, S1 gene sequencing identified subtler genetic variations, as demonstrated in sample D5, highlighting the complementary function of both methods. HRM is especially well-suited for routine surveillance and vaccine monitoring due to its benefits, which include its speed, affordability, and closed-tube workflow.

## Supplementary Information

Below is the link to the electronic supplementary material.

Supplementary Material 1

## Data Availability

The datasets used and/or analysed during the current study are available from the corresponding author upon reasonable request. All Sequence data generated in this study have been deposited in the NCBI GenBank database under the following accession numbers: N/3′UTR targets [LC731807, LC731808, LC731809, LC731810, LC731811, LC757451, LC757452, LC757453, LC757454, LC757455].and accession number for S1 targets [LC760462, LC760463, LC760464, LC760465, LC760466, LC760467].
