# Supplementary material for: Development of an HRM-qRT-PCR platform for fast and cost-effective genotyping of infectious bronchitis virus in Egypt
Source: Sci Rep. 2026 Apr 10;16:12053. doi: 10.1038/s41598-026-45311-9 (PMC13069100; doi:10.1038/s41598-026-45311-9)
Supplement: Supplementary file 1 — Supplementary Material 1 [file 41598_2026_45311_MOESM1_ESM.docx]

**Supplementar 1: IBV vaccine strains used as reference in this study**

| **References** | | **Commercial Name** | **Sources** |
| --- | --- | --- | --- |
| **IBV vaccine strains** | **Vac.1** | Ma5 | Nobilis (MSD) |
|  | **Vac.2** | Poulvac IB-Primer | Zoeitis |
|  | **Vac.3** | IBV 4/91 | Nobils (IMSD) |
|  | **Vac.4** | IBV 1212B(VAR II) | Mevac |

**Supplementary 2: Oligonucleotide primers used in the study of infectious bronchitis viruses in ﬁeld specimens**

| **Primer** | **Target/location** | **Sequence (5′–3′)** |
| --- | --- | --- |
| **All-F** | 3′UTR/26930-26948 | CAGCGCCAAAACAACAGCG |
| **Del1-R** | 3′UTR/27362-27344 | CATTTCCCTGGCGATAGAC |
